# Supplementary material for: Are carbon emissions trading and green financial instruments synergistic? -Comprehensive quantitative research based on content analysis
Source: PLoS One. 2024 Mar 7;19(3):e0298601. doi: 10.1371/journal.pone.0298601 (PMC10919601; doi:10.1371/journal.pone.0298601)
Supplement: S1 Data — (ZIP) [file pone.0298601.s001.zip › Data/地级市Z_F工作报告.zip!/╒π╜¡/╜≡╗¬╩╨/└┤╫╘╣1⁄2╓┌║┼í╛┬φ┐╦╩2╛▌═°í┐_2018.docx]

各位代表：

现在，我代表金华市人民政府向大会作政府工作报告，请予审议，并请市政协委员和其他列席人员提出意见。

一、2017年主要工作回顾

2017年是党的十九大召开之年，也是本届政府的开局之年。在省委、省政府和中共金华市委的坚强领导下，市政府深入学习贯彻习近平新时代中国特色社会主义思想，全面贯彻落实党的十九大精神，认真落实省第十四次党代会、市第七次党代会和市委七届二次全会决策部署，认真执行市人大及其常委会作出的各项决议，和全市人民一道，迎难而上、砥砺前行。全市实现地区生产总值3870亿元，增长6.5%；一般公共预算收入357.7亿元，增长8.3%；城乡居民人均可支配收入分别达到50653元和23922元，增长8.8%和9.3%，都市区建设迈出坚实步伐。

（一）勠力同心建设“三条廊道”，推进群城聚市。

廊道项目全面推进。加快综合交通廊道建设，突出轨道交通、高速公路、快速干线“三个闭合圈”，完成投资282亿元。金义东市域轨道交通项目全线开工、试验段提前竣工。杭金衢高速公路拓宽一期、义乌疏港高速公路建成通车，金兰中线、金武快速路进展顺利，义永快速路建设取得突破。义乌机场完成升级改造，横店通用机场投入使用。建成游埠、姚家两个航运枢纽。启动金义科创廊道建设，新增科创园区20个。金华科技城建立市县共建机制。与浙江师范大学开展全面战略合作，浙师大婺城科技园启动建设，金华理工学院获批筹建。推进浙中生态廊道建设，实施主廊道示范项目30个，完成投资484亿元，建成一批美丽城防、生态绿道、彩色林带和湿地公园。

共建氛围明显增强。合力共筑“同心圆”，实施都市区建设第二个三年行动计划，谋划实施重大项目50个。与中国城市规划设计研究院开展战略合作，启动编制金义都市区规划、新一轮城市总体规划。成功举办首届金华发展大会，签约重大产业项目46个、人才项目12个，成立金华海内外人才总会和婺商总会。建立金华江全流域水质考核奖惩机制。众志成城打赢“6·25”特大洪水抗击战。

城乡融合步伐加快。深化智慧城市、海绵城市、公交都市试点，推进古子城、多湖中央商务区等重点区块建设，基本实现重点区块城市设计全覆盖。加大城市有机更新力度，超额完成“三改一拆”任务，完成城中村拆迁238个、整治203个。小城市培育试点和小城镇环境综合整治工作扎实推进，磐安启动撤镇建街工作，全市城镇化率达到66.7%。金兰城际公交、BRT5号线开通运行，实现建制村“村村通客车”，市区公交分担率提高到21.4%。“三个标配”引领美丽乡村升级，金东农村生活垃圾分类、浦江美丽乡村建设经验入选“砥砺奋进的五年”大型成就展，新增省A级以上景区村庄205个。农村改革深入推进，消除集体经济薄弱村1198个。

（二）加快新旧动能转换，推进实业兴市。

“三去一降一补”扎实推进。坚决打破拖累高质量发展的坛坛罐罐，处置43家“僵尸企业”，淘汰149家企业落后产能，整治9959家脏乱差小作坊，减轻企业负担115.6亿元。实施防控金融风险“五大专项行动”，不良贷款率降至2.22%。积极对接多层次资本市场，新增上市企业5家、挂牌企业241家、直接融资302亿元，金融服务实体经济能力进一步提升。扎实推进永康、金华开发区供给侧结构性改革省级试点，“创客通”等7条经验全省推广。

五大千亿产业加快发展。信息经济综合评价得分居全省第二，网络零售额2346亿元、增长28%，互联网泛娱乐产业方兴未艾，省数字创意产业试验区获批建设，快递业务量居全国第四。菜鸟·金华电商园正式开园，浙江电信云计算中心投入运营，启动创建国家网络市场监管与服务示范区。先进装备制造、健康生物医药产业产值（收入）分别增长12.7%、10.1%，新能源汽车产业集群初具雏形。文化影视时尚产业产值（收入）增长10.2%。永康获评国家体育产业示范基地。旅游收入突破千亿元，迈入全省第一方阵，获评全国“厕所革命”综合推进先进市，新增浦江仙华山、磐安大盘山2个国家级风景名胜区。

重大项目加快落地。引进一批投资超20亿元的重大产业项目，捷孚传动、华灿光电等项目建成投产，爱旭太阳能、零跑汽车等项目加快建设，众泰福特新能源汽车、花园生物产业园等项目签约落户。完成固定资产投资2200亿元、增长11.2%，民间投资、服务业投资增速高于全省平均水平。

传统产业改造提升迈出新步伐。深入开展兰溪纺织、义乌服装、永康五金等传统制造业改造提升省级试点，获批立项“浙江制造”标准30个，新增国家级出口产业质量安全示范区3个，永康入围中国质量奖提名奖。新增中国驰名商标5个，阔别35年的“金华火腿”商标顺利回归。开展“关心关爱企业家、助企强企增活力”主题服务活动，深入实施小微企业三年成长计划，新增市场主体16.5万户、居全省第二，入围省“隐形冠军”培育库企业38家。实现建筑业总产值3472亿元，东阳建筑业总产值实现全国县级市九连冠。扎实推进农业供给侧结构性改革，新建省级粮食生产功能区8.8万亩，建设省级现代农业园区2个，新增省级美丽牧场69家。推进垦造耕地“双万工程”，新增耕地1.2万亩。武义、磐安获评全省休闲农业与乡村旅游示范县。

（三）以“最多跑一次”撬动各领域改革，推进创新强市。

“最多跑一次”跑出金华特色。在全省率先实现商事登记全程电子化和电子证照应用。实施企业投资项目“承诺制+标准地”改革，金东推出首块“标准地”。政府非税收入电子化收缴应用、“警医邮”服务、不动产登记集成服务、政务服务APP应用等便民举措获群众点赞。市政府新办事大厅投入使用，全面推行“一窗受理、集成服务”，“最多跑一次”事项实现率达到90%以上。

创新驱动力不断增强。加强科技创新，新认定国家高新技术企业254家、省科技型中小微企业679家、省级企业研究院15家，科技市场技术交易额增速居全省第三，第16届工科会成效显著，高新技术产业、战略性新兴产业规上增加值分别增长8%和9.4%。金华国家农业科技园区通过验收，入选省级特色小镇创建和培育名单3个、省级产业创新服务综合体创建名单2个。加强制度创新，启动实施国际贸易综合改革试点第三个三年行动计划，义乌宅基地“三权分置”试点成效显著。国家现代服务业综合试点深入推进，试点项目完成投资259亿元，服务业增加值占比达到53.4%。浦江“四个全面”等全市40多项国家和省部级改革试点扎实推进。

人才新政落地见效。出台“人才政策30条”，推行人才“金卡”服务，试行技能人才晋升工程师制度，新建院士专家工作站10家，实现“****”专家超百人、“双龙计划”项目超百个、人才资源总量超百万、人才企业产值超百亿。

（四）加快建设“三大通道”，推进开放活市。

国际商贸优势进一步巩固。外贸出口总额3311亿元，总量居全省第二。启动义乌国际贸易综合改革试验区建设。推进通关一体化、贸易便利化，80%以上货物实现“秒放”，推进“单一窗口”建设，报检量居全省第二。金义综合保税区封关运行。

“三大通道”不断拓展。加快建设陆上大通道，义新欧班列全年往返168列，市区开通中亚班列。规划建设“一带一路”捷克站，累计建成省级海外仓10家，数量居全省第一。加快建设海上大通道，“义甬舟”重大支撑性项目完成投资460亿元，海铁联运量增长200%，兰溪嘉宝获批内陆口岸进口转检业务。加快构建网上大通道，跨境网络零售出口占全省55.1%，义乌国际邮件互换局业务量居全国第四。成功举办第三届全球跨境电商大会。

对外交流合作深入开展。“走出去”与“请进来”并举，实际利用外资4.3亿美元、增长22.4%，新增对外总投资11.65亿美元，红狮集团积极布局“一带一路”产业基地，成为全国水泥行业“走出去”标杆。加快推进中欧、中捷、中加国际产业合作园建设。开展对外友好交往，举办两季海外名校学子走进金华古村落活动，新增国际友城7个。推进四省九方经济区合作，深入实施东西部扶贫和“山海协作”工程，全面开展与吉林四平对口合作，深化援疆援藏援青援川等工作。

（五）顺应人民美好生活需要，推进环境立市。

生态环境持续改善。全面落实中央环保督察要求，高质量办结信访交办件905件。认真贯彻《金华市水环境保护条例》，打赢剿劣攻坚战，实现“干流水质全达标、支流溪流灭Ⅴ类”，兰江将军岩出境断面水质均值达到Ⅱ类，为20年来最好水平。强化大气环境综合治理，细颗粒物（PM2.5）平均浓度降至42微克/立方米，空气质量改善幅度居全省第二。强化土壤环境综合治理，新增危废处置能力5.6万吨。启动国家级森林城市群创建，实现国家卫生城市、省级园林城市全覆盖。武义获评全球绿色城市，浦江获评国家级生态文明建设示范县。

人文环境不断提升。推进“信义金华”建设，发挥道德模范、“金华好人”示范引领作用，金华及东阳、义乌获全国文明城市创建提名资格。兰溪、东阳通过省教育基本现代化县评估。浙江师范大学行知学院兰溪校区建成招生。浙江广厦建设职业技术学院专升本、横店电影学院筹建列入省“十三五”高校设置规划。实施文艺精品工程，获全国精神文明建设“五个一工程”奖2项，婺剧《宫锦袍》入选国家重点扶持剧目。推进文化惠民工程，举办非物质文化遗产精品节目演出等系列文化活动。“金华发布”影响力全省第二。

社会环境不断优化。新增财力的70%以上用于民生支出，十方面民生实事项目全面完成。城镇新增就业10.4万人，基本养老保险、基本医疗保险参保率再创新高，实现大病医疗保险市级统筹，“病组点数法”支付方式改革取得积极成效。推行家庭责任医生签约服务，实现医联体对基层医疗机构一体化管理全覆盖，东阳开展医共体建设省级试点。实现城乡低保标准一体化。市第九届运动会成功举办，校园足球成为全省样板。保障性安居工程交付使用2万套。圆满完成村（社区）集中换届工作。乡镇（街道）“四个平台”全面建成运行，东阳入选首批全国农村社区治理实验区。整合提升8890便民服务平台。平安护航党的十九大，社会治安、信访维稳、安全生产、食品安全形势总体平稳，连续12年获评“平安市”，成功捧回“平安金鼎”。

全面发展老龄、残疾人和关心下一代等事业，支持工会、共青团、妇联等群团组织开展工作，加强民族、宗教、外侨、对台、统计、气象、民防、文物、档案、红十字会、地方志、仲裁、应急管理等工作。推进“七五”普法，做好农业普查和污染源普查。支持国防建设，开展庆祝建军90周年系列活动。

一年来，我们按照市委统一部署，扎实推进政府系统“两学一做”学习教育常态化制度化。加快法治政府建设，推进行政复议体制改革，成立市行政复议局。自觉接受人大法律监督和工作监督、政协民主监督，认真办理人大代表建议416件、政协委员提案494件。主动接受监察监督、司法监督、社会监督和舆论监督，加强审计监督。做好政务公开。强化“一岗双责”，加强作风建设，坚决惩处腐败行为。从严落实中央八项规定精神，“三公”经费连续6年下降。

各位代表，2017年发展成绩来之不易。这是习近平新时代中国特色社会主义思想和“八八战略”在金华生动实践的结果，是省委、省政府和中共金华市委正确领导的结果，是全市人民创业创新、共同奋斗的结果。在此，我代表市政府，向全市人民，向市人大代表、政协委员，向外来投资者、建设者表示崇高的敬意和衷心的感谢！向离退休老干部，向各民主党派、工商联、人民团体、无党派人士，向驻金人民解放军和武警部队官兵、中央和省属驻金单位，表示衷心的感谢！向关心和支持金华发展的港澳台同胞、海外侨胞和海内外朋友们，表示衷心的感谢！

我们也清醒地看到，我市发展不平衡不充分的突出问题尚未解决，政府工作还存在许多不足和挑战。主要有：都市核心区首位度仍然偏低，市域统筹能力亟待提升；高质量发展的基础还需不断夯实，新旧动能转换的步伐还需加快，地区生产总值增速没有完成预期目标；重大改革的政策措施仍需进一步落实；重大风险防范、低收入百姓增收、污染防治任务依然艰巨；民生领域还有不少薄弱环节；少数干部能力缺乏、不敢担当，形式主义、官僚主义新表现需要高度警惕。对此，我们一定高度重视，采取更加有力措施加以解决。

二、2018年目标任务和重点工作

2018年是贯彻落实党的十九大精神的开局之年，是改革开放40周年，是决胜全面建成小康社会、实施“十三五”规划承上启下的关键之年。2018年政府工作的总体要求是：高举习近平新时代中国特色社会主义思想伟大旗帜，全面贯彻党的十九大，十九届二中、三中全会和全国两会精神，认真贯彻省第十四次党代会、市第七次党代会和市委七届二次全会部署，坚定不移沿着“八八战略”指引的路子走下去，不忘初心、牢记使命，当好新时代金华答卷人，围绕“全面小康、浙中崛起”“走在前列、共建金华”目标要求，坚持和加强党的全面领导，坚持稳中求进工作总基调，坚持新发展理念，紧扣社会主要矛盾变化，按照高质量发展的要求，以供给侧结构性改革为主线，实施现代化都市区建设十大战略行动计划，促进经济持续健康发展和社会和谐稳定，为“两个高水平”和现代化都市区建设奠定坚实基础。

建议全市经济社会发展主要预期目标：地区生产总值增长7%左右，城乡居民收入、财政收入增长与经济增长同步，全员劳动生产率稳步提高，节能减排降碳确保完成省定任务，争取更高质量、更高效益、更好结果。

要实现以上目标，必须在五个方面加压奋进：在“高质量”上加压奋进。在更好和更强上聚焦发力，发挥金华籍人才较多的优势，锚定先进制造业、现代服务业，培育壮大新动能，提升转型旧动能，打造“双创”升级版，加快建设全省高质量发展的重要增长极。在“强改革”上加压奋进。坚定不移推动改革裂变、开放跨越，以“最多跑一次”改革为支点撬动全面深化改革，统筹抓好重大改革试点落地生根，全面融入“一带一路”建设，厚植开放通道、改革试点、市场大市优势，提升开放型经济发展水平，加快形成内陆城市开放发展的新格局。在“促共建”上加压奋进。对接融入大湾区大花园大通道大都市区建设，着力金义一体化、全域同城化、发展特色化，内建综合交通、金义科创、浙中生态“三条廊道”，外筑义新欧、义甬舟、跨境电商“三大通道”，共绘“同心圆”“八骏图”，加快建设组团式现代化都市区。在“抓攻坚”上加压奋进。努力跨越非常规的现阶段特有关口，坚决打好“三大攻坚战”，确保金融生态根本好转、政府债务安全运行，确保脱贫“消薄”任务全面完成，确保生态环境质量全面改善，为新时代现代化都市区建设扫清障碍。在“惠民生”上加压奋进。把人民对美好生活的向往作为奋斗目标，尽力而为、量力而行，推进基本公共服务均等化标准化，抓好事关群众切身利益的“关键小事”，加快提升城市品位、提升都市区软实力、提升社会治理水平，让发展更有温度、让幸福更有质感。

（一）聚焦高质量，让转型升级的步伐更加有力。

认真实施先进制造业和现代服务业双轮驱动战略行动计划，大抓实体经济，以数字经济为核心、以智能化为引领，打造高质量发展的现代产业体系。

加快培育壮大新兴产业。大力发展以新能源汽车、生物医药、光电子产业为重点的先进制造业，以网络经济、智慧物流、影视文化为特色的现代服务业，积极培育以量子科技、人工智能等为方向的重量级未来产业。抢抓数字经济变革的时间窗口，实施大数据发展行动，培育大数据、云计算、物联网产业，打造数据强市、“数字金华”，培育互联网乐乐小镇等数字经济集聚平台，争创国家数字文化产业试验区。加快横店影视文化产业集聚区建设。积极推进军民融合发展。

加快改造提升传统产业。认真落实中国制造2025战略规划，以纺织服装、五金等八大传统制造业为重点，加快实施“机器人+”、企业上云、标准提升、品牌创建等十大行动，推进永康“浙江制造”标准集中申报试点，全市新增“浙江制造”标准50个、市“两化”融合示范重点项目100个、上云企业1万家，培育产业创新服务综合体9个。实施小微企业提质行动，新建小微企业园50个、标准厂房200万平方米，新增“个转企”1200家、“小升规”300家、省“隐形冠军”培育库企业40家。积极“去产能”“降成本”，处置30家“僵尸企业”，淘汰100家企业落后产能，整治2000家脏乱差小作坊，为企业减负100亿元以上。经营好“金华火腿”等老字号品牌，推动历史经典产业改造提升。发展会展经济，全面开展“放心消费在金华”行动，推进商品交易市场创新提升。

深入开展“优资源、强平台、促发展”集中攻坚活动。加快打造一批“万亩千亿”重大工业平台、城市建设平台、特色农业平台，拓展或新增重点产业平台20个以上，拓展产业发展空间2万亩以上。启动“海关特殊监管区+开发区”整合工作，推动现有工业功能区、产业园、创业孵化基地等改造提升、整合转型。优化存量配置，消化批而未供土地2万亩，低效用地再开发7000亩。

切实抓好招商引资“一号工程”。发挥金华籍企业家、科学家较多的优势，开展招大引优“六个一”活动，大力推进市县长项目工程，加大产业链招商、精准招商力度，着力培育新产业、新动能、新增长极，引进投资超20亿元重大产业项目20个，其中超50亿元6个。落实“投资新政”，高新技术产业投资、生态环境和公共设施投资均增长15%以上，交通投资、民间投资均增长10%以上，市县长项目工程落地率50%以上。

认真实施创新驱动发展战略行动计划，以科技创新引领产业创新，增强高质量发展的动力。

培育创新主体。深入实施科技企业“双倍增”计划，设立科技投资基金10亿元以上，支持企业加大科技投入，研发经费相当于生产总值的比例提高到2.15%左右，力争新增国家高新技术企业120家、省级科技型中小微企业480家。

积极推进科技成果产业化。实施发明专利产业化项目100项，力争实现技术交易额20亿元，办好第17届工科会。加大知识产权保护运用力度，万人发明专利拥有量达到9件以上，争创国家知识产权示范城市、国家创新型试点城市。

加快建设人才强市。树立“人才是第一资源”理念，全面落实“人才新政”，全面优化“双创”生态，实施“****”“双龙计划”“婺星回归计划”，新增院士工作站3个、博士后工作站3个，引进高层次人才2000人。深化“八婺英才”培养体系，抓好“草根”人才评价、工程师直评等工作，壮大创新型科技人才、经营管理人才和高技能人才三支队伍。弘扬劳模精神和工匠精神，建设知识型、技能型、创新型劳动者大军。

认真实施乡村振兴战略行动计划，扎实推进现代化“和美乡村”建设，夯实高质量发展的基础。

着力增强农业农村实力。实施产业提质行动，深化农业供给侧结构性改革，加大农产品公共区域品牌创建力度，探索组建乡镇国有土地流转公司，提升农业“两区”建设水平，建设省级现代农业园区3个、绿色农产品基地1万亩、省级美丽牧场50家，培育市级以上农业龙头企业10家、示范性家庭农场30家。全面落实粮食安全责任制，坚决守住耕地红线。构建农村一二三产业融合发展体系，加快发展农产品加工业，全域发展休闲农业与乡村旅游，建设休闲观光农业园区10个。

着力释放农业农村活力。加快农村土地所有权、承包权、经营权分置改革，深化农村集体经济股份合作改革，推进宅基地所有权、资格权、使用权分置改革，提高农村资源要素配置效率。完善农产品流通体系，加强面向农户的社会化服务，支持农合联与农民专业合作社联动发展。实施乡村治理创新行动，发挥乡贤作用，健全自治、法治、德治相结合的乡村治理体系。实施惠民富民行动，加强农村实用人才、新型职业农民培训，提升农村公共服务水平。

着力彰显农业农村魅力。紧扣建设大景区大花园目标，实施生态修复和乡风文明提升行动，持续推进“拆违治危”“四边三化”、小城镇环境综合整治，深化“厕所革命”，加强农村人居环境整治，加大传统村落保护力度。系统推进“和美乡村”建设示范镇、精品村和历史文化村落创建，新增省A级景区村庄300个，其中3A级30个以上。高水平建设“四好农村路”850公里。

（二）扛起改革旗，让体制机制的优势更加凸显。

认真实施全面深化改革战略行动计划，以“最多跑一次”改革为突破口，勇立新时代的改革潮头。

深化“最多跑一次”改革。以标准化引领业务流、信息流、审批流整合，所有民生事项和企业事项实现“一次办结”，其中80%以上开通网上办理，50%以上的民生事项实现“一证通办”，推进涉企事项“一码通用”。加快“一窗受理、集成服务”向基层延伸，更多事项实现“就近跑”“零上门”。深化“标准地+承诺制+代办制”改革，全面实施“区域能评、环评+区块能耗、环境标准”，实现企业投资项目开工前审批全流程最多跑一次、最多100天。深化商事制度改革，全面推进“证照分离”，统筹推进“多证合一、证照联办”。市区要按照“最多跑一次”的要求，完善管理体制和工作机制。

深化要素市场化配置改革。新批工业用地的50%按照“标准地”制度供地。深化“亩均论英雄”改革，全面整治亩均税收1万元以下的低小散企业。优化财政支出结构，强化零基预算，加强绩效管理，实行专项资金使用动态监管。弘扬企业家精神，保障企业家合法权益，鼓励社会资本进入医疗、养老、教育、文化、体育等领域。

深化国资国企改革。坚持市场化改革方向，重组市属国有企业，组建一批资产规模较大、主营业务突出、市场化运营能力强的国有企业集团，承担重大基础设施、重要民生项目的投融资建设与运营管理。推动市、县国有企业相互参股。

推动改革系统集成、落地见效。提升推广“义乌发展经验”“后陈经验”、领导干部下访等经验。建立改革试点管理台账，深化国际贸易、现代服务业、影视文化等国家和省部级改革试点，创造更多“金华经验”。

认真实施开放发展战略行动计划，以“一带一路”为统领，构建对外开放新格局。

共建陆上大通道。聚全市之力培育发展义新欧班列，力争往返400列，其中中亚班列100列，联动推进常态化、市场化运行。积极布局海外仓，不断扩大国际“友城圈”，加快建设“一带一路”捷克站。提升铁路口岸、保税口岸功能，发展航空物流，培育内陆口岸经济。

共建海上大通道。抓好“义甬舟”重大支撑性项目建设，培育外向型产业群、高新产业带和物流集聚区。加快建设金甬铁路和浙中公铁联运港，发展公铁联运、海铁联运、多式联运。推进“港港联动”，实现与宁波舟山港“同关同检、同港同策”。

共建网上大通道。推进跨境电商、实体市场、产业集群融合发展，加快金义跨境电商产业园和跨境直销中心建设，完善跨境电商基础设施，扩大跨境电商出口领先优势。争创中国（义乌）跨境电子商务综合试验区，开展跨境电商保税进口业务，继续办好全球跨境电商大会、世界电商大会。

扩大优进优出规模。做强市场采购出口、高品质产品出口、服务贸易出口，继续办好义博会等重点展会，组织参加境内外展会100场，争创国家服务外包示范城市，力争对“一带一路”沿线国家出口超1500亿元。积极对接中国国际进口博览会，扩大先进设备和优质中间产品进口。

扩大经贸文化交流合作。加大利用外资工作力度，建设国际产业合作园。支持企业集团式、产业链式走出去，收购专利技术、设立研发机构、建设营销网络和生产物流基地，培育本土跨国公司。办好中非智库论坛第七届会议、中非博览会。深化海外名校学子走进金华古村落活动。积极参与长三角区域、四省九方经济区合作互动和对口帮扶支援工作。

（三）共建都市区，让浙中崛起的势头更加强劲。

认真实施全域同城化战略行动计划，以“三条廊道”为主平台，研究“统”的文章、加大“联”的力度、增强“聚”的效应，加快提升都市区能级。

加快共建综合交通廊道。围绕“三个闭合圈”建设，推进交通建设大会战，完成投资300亿元以上。扎实推进金义东市域轨道交通项目，开展二期前期工作，完成轨道交通闭合圈规划。开工建设义东高速公路东阳段，尽快形成高速公路闭合圈。加快建设金武快速路、金兰中线和永武四线，提升改造东永城际快速路，新改建干线公路26条，建成美丽公路590公里，加快形成快速干线闭合圈。统筹内畅外联，加快推进金台、金建、杭温铁路和建金、杭绍台高速公路建设，开工建设杭金衢高速公路拓宽二期工程，积极推进衢江航运开发。

加快共建金义科创廊道。全面融入沪嘉杭金科创走廊，积极创建国家高新区。金华科技城完成“四横二纵”路网建设，积极引进高等院校和科研机构，年内落地科研机构5家以上，力争各县（市、区）都有项目落地。规划建设“****”产业园，推进义乌信息光电、永康现代农业装备等省级高新区和县域科创平台建设。

加快共建浙中生态廊道。认真实施绿色发展战略行动计划，以生态为基、水系为脉、廊道为要、文化为魂，打造独具特色的浙中大花园。加大“三大节点、十大板块”建设力度，完成投资360亿元以上，规划建设服务区驿站15个，新建改造彩色林带90公里以上，实现金义主轴廊道美丽城防建设全面启动、滨水绿道全面贯通、各县（市、区）均有示范区块。加快建设全域旅游目的地，推进金华山、东阳花园村、武义牛头山等创建5A级景区，加快兰溪古城保护开发，推出精品旅游线路10条以上，力争旅游收入增长12%。

认真实施金义一体化战略行动计划，不断增强金义主轴集聚辐射带动力。

大力提升金义主轴聚合度。着力规划一体化，推进“多规合一”，统筹协调重大区块、重大平台、重大项目建设。着力交通一体化，加快建设城际快速路网，开工建设全方位、高水平、快通达的金义中央大道，推进深层次的互联互通。着力开放一体化，共建国际贸易综合改革试验区，推进金义综合保税区和义乌B型保税物流中心一体运营、联动发展。着力公共服务一体化，在资源共享、服务同城上取得突破，加密城际快速公交。金义都市新区要朝着“十年立新城”的目标，拉高标杆、埋头苦干，发挥战略支点作用。

大力提升都市区核心区首位度。市区要有市区的担当，核心区要有核心区的能级。市区要加快发展，以提升核心区综合能级为目标，加快推进“十大区块、十大工程、十大项目”，建设浙中西教育中心、文化中心、医学中心，增强服务带动周边县市发展能力。义乌要勇立潮头、勇当标兵，全面实施市场赋能、改革引领、开放升级等行动，加快市场转型、产业转型、城市转型，高水平建设世界小商品之都。

推动区域联动协调发展。各县（市）要在都市区发展大局中找准定位，优势互补、相互借重、互促共进，实现高质量特色发展。兰溪要加快振兴老工业基地，推进古城焕发新春，打造产业振兴示范区、古韵风情旅游地、品质活力幸福城。东阳要努力把文化优势、人才优势转化为发展优势，打造影视旅游名城、经济文化强市。永康要发挥制造业优势，强化科技创新，加快向智造强市跨越，打造先进制造业基地。浦江要深化改革、加快产业创新，推进“美丽浦江”向“繁荣浦江”转变。武义要加快绿色转型、美丽富民，推进全域化旅游，打造温泉名城、养生胜地。磐安要推进绿色发展和品质山城建设，打造江南生态养生源、身心两安“大花园”。

（四）打赢攻坚战，让全面小康的基础更加稳固。

打赢防范化解重大风险攻坚战。强化统筹协调，深化防控金融风险“五大专项行动”，力争不良贷款率控制在2%以内，实现金融生态的根本好转。深化金融改革，发挥金融对实体经济支撑作用，新增贷款500亿元以上。实施企业对接多层次资本市场“尖峰行动”，新增股份制企业100家、上市挂牌企业60家，实现直接融资360亿元。加强政府债务管控，实施五年化债计划，确保政府债务安全运行。保持基本养老保险基金支付能力总体稳定。

打赢低收入百姓增收攻坚战。就业培训、技术帮扶与下山脱贫相结合，因户施策、精准扶贫，低收入农户增收10%以上。完善医保体制、助学机制和灾害救助体系，基本消灭因病致贫、因学致贫、因灾致贫现象。深化“2+1结对帮扶”行动，大力发展物业经济，全面消除集体经济薄弱村。支持武义、磐安绿色发展，支持民族乡镇加快发展。

打赢污染防治攻坚战。坚决完成中央环保督察问题整改，加大环保督查力度，坚决防止环境污染问题反弹回潮。打好蓝天保卫战，制定实施三年行动计划，深化挥发性有机物（VOCs）治理，推进扬尘污染防治，二氧化碳强度降低4.6%以上，空气质量优良率达到78%以上。打好剿劣巩固战，深化河长制，创新湖长制，启动“污水零直排区”和“美丽河湖”创建，高质量建设污水处理设施，新增污水日处理能力7.8万吨。打好垃圾治理攻坚战，实施城乡垃圾源头减量、回收利用、处置能力提升等专项行动，扎实推进市第二生活垃圾焚烧发电项目，加快建立垃圾分类处置系统，打造农村垃圾分类全国标杆。打好土壤污染防治战，开展土壤污染源清查与防治，抓好污染土地的管控、修复和安全利用，污染地块安全利用率超91%。推行生态环境损害赔偿制度和市域上下游生态补偿机制，加强生态系统保护和修复。

（五）织好幸福网，让人民群众的生活更加美好。

认真实施软实力提升战略行动计划，完善基本公共服务体系，努力增强人民群众获得感、幸福感。

提高就业质量和社会保障水平。实施积极就业政策，深化“青年创业谷”计划，做好重点人群就业帮扶，城镇新增就业8万人以上，城镇登记失业率控制在3.5%以内。实现基本医疗保险市级统筹，推进医疗和生育保险合并实施，深化在职职工住院医疗互助保障。支持医养融合机构健康发展，完善居家养老服务体系。大力发展社会救助、社会福利、慈善事业，保障妇女儿童合法权益，推进残障人康复工程和无障碍设施建设。

打响“学在金华”品牌。坚持教育优先发展，实施学前教育新一轮三年行动计划，推进城乡义务教育优质均衡发展，积极消除“大班额”，完成薄弱学校改造项目22个。开展课外培训机构专项治理，切实为中小学生“减负”。推动高中特色化发展。深化职业教育产教融合、资源整合，推行“3+2”中高职衔接办学模式。加快建设高等教育强市，积极引进知名高校，大力支持在金高校发展，加快筹建金华理工学院。发展特殊教育、继续教育和网络教育。加强师资队伍和师德师风建设。

打响“健康金华”品牌。深化“双下沉、两提升”和分级诊疗制度，支持医联体、县域医共体发展。加大学科建设力度，加快形成以三甲医院为龙头、等级医院为骨干、专病中心为特色的医疗体系。提高基层医疗机构服务能力，加强疾病防控，完善妇幼健康计生服务保障，构建“20分钟医疗卫生服务圈”。发展中医药事业。实施食品安全战略，积极创建国家食品安全城市。加强基层体育场地设施建设，广泛开展全民健身活动。

打响“文化金华”品牌。开展“迎亚运、创文明”主题活动，启动亚运分村及运动场馆建设，扎实开展全国文明城市创建活动。深入实施“文化金华”建设十大工程，完善公共文化服务体系，统筹推进乡镇文化站和村综合文化服务中心建设。加大历史文化名城建设力度，保护历史文化街区，创建省级文化创意街区，发挥历史文化名人效应，加大“上山文化”研究宣传力度。加强文物保护和非物质文化遗产保护利用，振兴婺州窑等传统工艺，推进婺剧传承发展。以“八婺好家风”行动为引领，倡导移风易俗，传承优秀传统文化，培育文明乡风、良好家风、淳朴民风。

打响“宜居金华”品牌。推进精品城市建设，古子城代表着城市的历史，老火车站承载着市民的记忆，多湖中央商务区展示着金华的未来，要有品位、出形象、成亮点。各县（市）要完善优化一批体现各地特色的博物馆、规划馆、图书馆等城市“标配”。合理引导市场预期和购房行为，促进房地产市场平稳健康发展。深化住房制度改革，完善公共租赁住房保障制度，开展义乌租售同权试点。培育发展小城市、中心镇，深化无违建创建，加大城市有机更新力度，完成拆违1200万平方米、“三改”1700万平方米，其中城中村改造1000万平方米。

认真实施社会治理水平提升战略行动计划，共建共治共享美好家园，不断提升群众安全感。

深化“平安金华”建设。总结提升平安护航党的十九大等经验，做好信访工作，提升矛盾纠纷调处和风险排查处置水平。健全社会公共安全体系和社会治安防控体系，推进“雪亮工程”建设，坚决扫黑除恶，依法打击各类违法犯罪活动。完善安全生产责任制，坚决遏制重特大安全事故。保障网络安全，维护国家安全。推进地质灾害隐患点“三年清零”和农村危旧房治理。提升防汛防台、防灾减灾救灾、应急管理和预警预防能力。加强社会心理服务体系建设，培育自尊自信、理性平和、积极向上的社会心态。

完善社会治理体系。推进“基层党建+基层治理创新”，深化“四个平台”建设。做好“七五”普法，推进“全科网络”和民主法治村（社区）建设，加强社区治理，发挥社会组织作用，推动社会治理重心向基层下移。深化“双随机、一公开”监管，强化失信“黑名单”惩戒机制，健全社会信用体系。支持国防建设，争创“省级双拥模范城”六连冠。

各位代表，我们将继续按照“群众提、代表定、政府办、人大评”的要求，认真办好十方面民生实事。

三、切实加强政府自身建设

以党的十九大精神为指引，以“两强三提高”为目标，坚持“政府的定位是干事，核心要求是落实，优秀品质是担当，检验标准是干成事”，紧扣发展、狠抓落实，全力打造职能科学、结构优化、廉洁高效、人民满意的服务型政府。

（一）维护核心，忠诚干事。牢固树立“四个意识”，坚定不移维护核心、忠贞不渝紧跟领袖，在政治立场、政治方向、政治原则、政治道路上同以习近平同志为核心的党中央保持高度一致。坚持把学懂弄通做实党的十九大精神作为首要政治任务，大力弘扬红船精神，开展“不忘初心、牢记使命”主题教育，落实全面从严治党要求。

（二）服务为民，务实干事。坚持以人民为中心的发展思想，完善领导干部联系群众制度，用好政务咨询投诉平台，把更多的财力向民生领域倾斜。从人民群众关心的事情做起，从让人民群众满意的事情做起，高质量开展大调研活动，高质量制定实施政策，高质量履行经济调节、市场监管、社会管理、公共服务、生态环境保护等职责。

（三）雷厉风行，高效干事。推行政府工作清单管理、挂图作战、擂台比拼，变“推着干”为“比着干”。探索“即决策、即执行、即监督、即反馈”的高效闭环工作机制，让“马上就办”成为常态。建立执行台账制度，让实干担当成为自觉。推广运用“微政务”、大数据和移动办公系统，推进政府治理数字化转型。实施容错免责机制，鼓励干部大胆探索、改革创新。

（四）严以用权，依法干事。加强法治政府建设，增强宪法观念，坚持以宪法立规矩、立秩序、立诚信，依照宪法法律履行职责，不断提升依法行政、为民服务的能力和水平。完善向同级人大报告、向同级政协通报制度，发挥法律顾问作用，坚持常务会议学法等制度。支持做好地方立法工作。自觉接受人大依法监督、政协民主监督和监察监督、司法监督，切实加强政府内部层级监督和审计监督，开展领导干部自然资源资产离任审计。深化政府机构改革，坚决破除制约市场在资源配置中起决定性作用、更好发挥政府作用的体制机制弊端，形成职责明确、依法行政的政府治理体系，增强政府公信力和执行力。加大政务公开力度，让权力在阳光下运行。

（五）清廉为本，干净干事。牢记作风建设永远在路上，严格落实中央八项规定精神，关注“四风”问题新表现新动向，在反对形式主义、官僚主义上下更大功夫，持之以恒正风肃纪。深化机关效能革命，强化责任担当，大力整治慵懒散，着力破解“为政不为”问题。以《中华人民共和国监察法》颁布实施为契机，加强廉政教育，认真开展“三清理一规范”专项行动，深入推进政府系统反腐败斗争，打造清廉政府。坚持以正服人、以信立人、以诚待人，努力营造创业创新、崇廉尚实、风清气正的干事氛围。

各位代表，幸福都是奋斗出来的！让我们更加紧密地团结在以习近平同志为核心的党中央周围，在习近平新时代中国特色社会主义思想指引下，紧紧抓住大有可为的历史机遇期，在省委、省政府和中共金华市委的坚强领导下，聚全民之力、扬奋进之帆，当好新时代金华答卷人，为高水平全面建成小康社会、高水平全面建设社会主义现代化、高水平全面建设现代化都市区而不懈奋斗！
